# Neutron Lifetime Experiment Based on an Accordion-Like *UCN* Storage Volume Coated With “Low Temperature Fomblin”

**DOI:** 10.6028/jres.110.052

**Published:** 2005-08-01

**Authors:** B. Yerozolimsky, A. Steyerl, O. Kwon, V. Luschikov, A. Strelkov, P. Geltenbort, N. Achiwa, A. Pichlmaier, P. Fierlinger

**Affiliations:** Harvard University, Cambridge, MA, USA; University of Rhode Island, Kingston, RI, USA; Joint Institute for Nuclear Research, Dubna, Russia; Institut Laue Langevin, Grenoble, France; Osaka University, Japan; Paul Scherrer Institut, Villigen, Switzerland

**Keywords:** neutron lifetime, ultracold neutrons

## Abstract

A new type of per-fluorinated polymer, “Low Temperature Fomblin,” has been tested as a wall coating in an ultracold neutron (UCN) storage experiment using a gravitational storage system. The data show a UCN reflection loss coefficient *η* as low as ≈ 5 × 10^−6^ in the temperature range 105 K to 150 K. We plan to use this oil in a new type of neutron lifetime measurement, where a bellows system (“accordion”) enables to vary the trap size in a wide range while the total surface area and distribution of surface area over height remain constant. These unique characteristics, in combination with application of the scaling technique developed by W. Mampe et al. in 1989, ensure exact linearity for the extrapolation from inverse storage lifetimes to the inverse neutron lifetime. Linearity holds for any energy dependence of loss coefficient *µ*(*E*). Using the UCN source at the Institut Laue Langevin we expect to achieve a lifetime precision below ±1 s.

## 1. Introduction

Particle decay data indicate that the Cabibbo-Kobayashi-Maskawa matrix may deviate from unitarity (presently at the 2.7-sigma level [1]). This question depends critically on the up-down quark mixing amplitude *V*_ud_, which is determined most sensitively by the neutron life-time *τ*_n_ and the neutron decay asymmetry coefficient *A*. A reliable, precise value of *τ*_n_ will also help to refine models of astrophysics [2] and cosmology [3]. The current world average is *τ*_n_ = (885.7 ± 0.8) s [4]. We propose a new *τ*_n_ measurement with a precision below 1 s, using UCN storage. Wall losses are minimized by the use of “low-temperature Fomblin,” and the notoriously difficult extrapolation from storage lifetimes to *τ*_n_ is made more reliable by the novel use of an accordion-like storage vessel. In this system, the surface area and its distribution over height remain constant while the volume is changeable in a wide range. Combination with the scaling technique of Mampe et al. [5] ensures that the extrapolation function becomes strictly linear for any shape of UCN spectrum in the trap and for any energy dependence of reflection loss coefficient *µ*. Moreover, no correction for gravity is required. These unique features distinguish this method from all previous *τ*_n_ experiments based on UCN storage in material traps [5–10].

## 2. Basic Considerations

In UCN-storage based *τ*_n_ experiments it is crucial that any non-decay loss due to wall collisions, gaps and the residual gas are reliably subtracted from the total loss rate. In the elementary theory of wall reflection loss the interaction with the wall atoms is described by a step-function barrier determined by the optical (or mean Fermi) potential *U*-i*W*. For an isotropic UCN distribution the mean loss probability per bounce is given by *µ*(*E*) = 2*η*{(*U/E*)arcsin(*E/U*)^½^−[(*U−E*)/*E*]^½^}, where *η* = *W/U* and *E* is the neutron kinetic energy at the impact point. The wall loss probability per second is

$${\tau_{w}}^{-1}=<v\mu \left(E\right)>$$ (1)

where *ν* is the wall collision rate for a UCN and the average is taken over the UCN spectrum and trap surface. Neglecting gravity, *ν* = *v/λ* can be expressed by the mean UCN velocity *v* and the mean free path *λ* = 4*V/S*. *λ* is independent of *v* and determined only by the total trap surface *S* and volume *V*. As shown in [5] this gas-kinetic result is also valid under gravity provided the trap geometry has a horizontal plane of symmetry (as in the “accordion-trap” discussed below) and all UCN have enough energy to reach the highest point(s) in the trap. For gravitationally bent paths we use the straightforward definition 1/*λ* = total wall collision rate *Nν* divided by the volume-integrated UCN flux Φ in the trap (see Sec. 4).

However, gravity does induce an important difference. For our trap geometry, the total UCN number *N* is not exactly proportional to volume *V*, even for identical spectra. As a consequence we will plot storage data vs *ν*, not 1/*λ*, to obtain a linear dependence.

If we are sure that a single constant (*η*) can be factored out of the function *µ*(*E*), Eq. (1) may also be written in the form *τ*_w_^−1^=*ηγ*, where *γ* is defined as *γ* = <(*v/λ*)(*µ/η*)>, averaged over spectrum and surface. However, this is strictly justified only for a smooth, uniform wall without surface contamination and/or microstructure (cracks, roughness etc.), which can be represented by a potential step function.

In an experiment we measure the numbers *N*(*t*_1_), *N*(*t*_2_) of UCN counted after storage times *t*_1_ and *t*_2_. Although for a broad UCN spectrum the decay curve is nonexponential one can define a mean storage lifetime for time interval *t*_1_, *t*_2_ in the form

$$\tau_{st}=\left(t_{2}-t_{1}\right)/\ln\left[N\left(t_{1}\right)/N\left(t_{2}\right)\right]$$ (2)

with *τ*_st_^−1^ = *τ*_n_^−1^ + *τ*_w_^−1^ + other losses.

Three different methods have been used to extract *τ*_n_ from storage experiments.

- In Refs. [5,6,10], the vessel geometry was changed to vary *λ* and extrapolate the measured dependence *τ*_st_^−1^(*λ*^−1^) to *λ*^−1^ → 0.
- In Ref. [7], the storage lifetime was measured for different intervals of UCN energy, corresponding to different mean *γ*-values, and the dependence *τ*_st_^−1^(*γ*) was extrapolated to *γ* → 0.
- In Ref. [9], storage lifetimes were measured together with the flux of UCN thermally up-scattered at the trap walls. This provided a further handle on the separation of wall losses from beta-decay.

Restricting the discussion to the extrapolation methods (a) and (b), the accuracy and reliability of the *y*-axis intersection is determined by several criteria:

- First of all, the reliability of the extrapolation law *τ*_st_^−1^(*λ*^−1^) or *τ*_st_^−1^(*γ*) (or e.g., *τ*_st_^−1^(*ν*)) used to bridge the gap *Δ*_1_ from (*λ*^−1^)_min_ or *γ*_min_ (or similar *x*-variables) to zero.
- The maximum *x*-range *Δ*_2_ between (*λ*^−1^)_min_ and (*λ*^−1^)_max_ (or similar) accessible to the experiment. If the dependence is linear and the statistical uncertainties are constant, the precision of the fitted *y*-intersection (*τ*_n_^−1^) is determined by the ratio *Δ*_2_/*Δ*_1_, and independent of the size of the gap *Δ*_1_ (the distance of *τ*_st_ from *τ*_n_).
- In addition to the experimental *y*-errors the uncertainties of calculated mean *x*-values like *γ* must be taken into account [11]. They include spectral and model uncertainties, most critically those related to the assumption of a step-function potential for the wall.

Method (a) ensures a linear dependence *if* all *τ*_st_ data are obtained with the same UCN energy spectrum at equivalent times of a storage cycle, so that only *λ* changes when the trap geometry is changed. The linearity can be checked for the experimental data, and the reliability of this test will also improve with increasing *Δ*_2_/*Δ*_1_. To ensure identical spectra in storage measurements with different *λ*, Pendlebury, Mampe et al. developed a scaling procedure where all time intervals *Δt* are chosen proportional to *λ* [5]. In this case, the total number of wall collisions in equivalent time intervals is the same, and therefore the spectra and spectral changes are practically the same for cycles with different *λ*-values. In Ref. [5], only small corrections were needed to take into account the “loading effect” (essentially the role of β-decay during trap loading and emptying) and gravity. Both corrections are essentially reduced for the system described below.

Method (b) relies on the validity of the assumed energy dependence of the wall loss. The calculation of *γ*-values is directly based on the step function potential model for the wall. Experiments show that for low-absorbing materials like per-fluorinated polymers at low temperature or solid oxygen the loss coefficient is significantly higher than calculated for a clean surface [12]. Moreover, in a 10^−5^ mbar vacuum at ≈120 K a clear deterioration of *τ*_st_ by several seconds was observed over time periods of hours to days [11], possibly due to surface contamination from the residual gas. Besides, calculation of the *γ*-values required for the energy extrapolation method depends on the UCN spectra. Therefore all spectral changes over a cycle must be known, including Doppler shifts due to trap rotation, and this is a difficult experimental task.

These considerations appear to favor method (a) in terms of reliability. Therefore the present proposal is based on method (a) with the additional benefit provided by a very-low-loss wall coating and by the bellows system used, which allows a large dynamical range (*Δ*_2_/*Δ*_1_). while the surface area is kept constant.

## 3. Features of the “Accordion System”

The new type of “low temperature Fomblin” (LTF) used for wall coating is a fluoro-polymer of composition CF_3_O(CF_2_O)*_n_*(CF_2_CF_2_O)*_m_*(OCF_2_CF_2_O)*_k_*CF_3_ with *n* = 30.3, *m* = 1.5, *k* = 0.2 [13]. It consists only of the low-absorbing elements C, F, and O, like ordinary Fomblin, but has an 80 K lower solidification temperature of ≈ 150 K. Thus it can be used in liquid form at low temperatures where losses due to inelastic scattering [12] and quasi-elastic scattering [14,11] are strongly reduced. In direct UCN storage measurements using method (b) we obtained *η* ≈ 5 × 10^−6^ in the temperature range 105 K to 150 K. This is the lowest wall loss coefficient reported so far but subject to uncertainty. Since no reliable extrapolation to *τ*_n_ seemed possible in these experiments, the *η*-value was calculated from the *τ*_st_^−1^ vs *γ* slope assuming intersection at the world average value *τ*_n_ ≈ 886 s. The largest storage lifetime obtained in these experiments was 872 s, which is < 2 % away from 886 s [11].

**Fig. 1:**
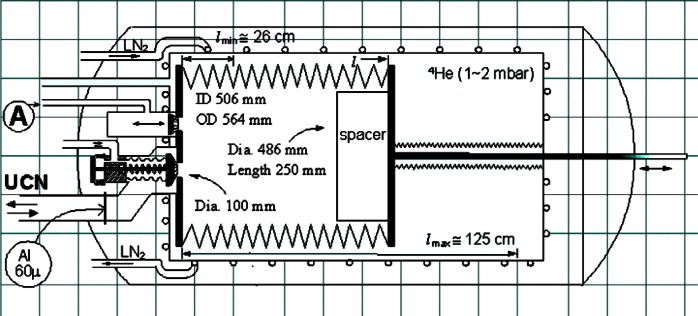
Schematic view of the “accordion” system. It contains a UCN trap whose surface area remains constant while the volume is changeable by a factor 27. The inner surface will be coated with “Low Temperature Fomblin” at temperatures in the range from 100 K to 220 K to provide a low-loss UCN storage system for a neutron lifetime measurement.

The new system, shown schematically in Fig. 1, uses trap size variation as in method (a). A 56 cm OD bellows with stretched length 125 cm is fitted into a horizontal cylindrical vacuum chamber. The system allows gap-free volume changes by a stepper motor moving with precision ≈ 0.01 mm. The wide volume range (and therefore also of *λ* and *ν*) by a factor >25 is achieved by insertion of a “spacer” which reduces the minimum usable separation of the vertical walls to ≈1 cm. A prominent feature of this device is the absence of changes of wall area and of its distribution over height. As shown below, this is an essential condition for straight-line dependence of *τ*_st_^−1^ on *ν*, independently of the energy dependence of reflection loss as well as of the UCN spectrum. An Al transmission foil (Fig. 1) provides a low-energy cutoff such that all admitted UCN are able to reach the roof.

The interior storage volume surface will be coated with LTF oil at the measurement temperature, which can be chosen in the liquid or solid range. The oil is condensed from vapor transported from a heated reservoir in low-pressure He gas (feature *A* in Fig. 1) and the coating is easily refreshed. Uniformity of surface temperature is achieved by embedding the “bellows volume” in a secondary vacuum vessel kept at a temperature variable between ≈100 K and 200 K. The thermal contact can be improved by low-pressure He gas.

Fig. 1 shows the principle of highly leak-tight UCN and oil vapor shutters. Residual leakage can be checked with helium gas.

“Scaling” will be used for trap loading, storage and emptying, i.e., all time periods will be proportional to volume *V*, and therefore strictly ∼*λ*.

## 4. Calculations

Details of the analysis of the “accordion-system” have been presented elsewhere [5]. The following assumptions were made.

- The initial spectrum of UCN entering the system is a Maxwell spectrum cut from above by the critical energy for LTF at ≈150 K (= 120 cm in units of *E/mg* with the neutron mass *m* and gravitational constant *g* [11]). From below there is a smooth cutoff due to Al foil transmission. Referring all UCN jump heights to the trap center at *h* = 0, the energy range of stored UCN is 30 cm < *h*_o_ < *h*_cr_ with *h*_cr_ = 120 − OD/2 = 92 cm, where OD is the outer bellows diameter.
- A loss coefficient *η* = 5 × 10^−6^ was assumed but this value and the associated model of a potential-step wall affect only the calculated storage lifetimes but have very little influence on quantities like collision rate or trap loading/emptying time constants.
- Transmission times and transmission losses between UCN valve and source or detector were neglected.
- For given height, the UCN spectra and densities were assumed to be uniform over the lateral trap extension at all times, even during trap loading and emptying. We plan to use Monte Carlo simulations to check the validity of this assumption.
- In most calculations a long storage time was used to make sure that averaging of loss rates (as in Eq. (2)) and of *ν* or similar quantities is justified even for long storage periods. For the largest volume *V*_max_ (fully stretched bellows) we chose: for loading *t*_f_ = 200 s; for storage *t*_1_ = 300 s (short storage time), *t*_2_ = 2300 s (long storage time), and for emptying *t*_e_ = 150 s.
- For smaller accordion volumes all times were reduced by the factor *λ*_max_/*λ* = *V*_max_/*V*.
- The interior trap surface is constant.
- Gravity was taken into account.
- UCN loss by leakage through the closed valves was neglected. It adds to the reflection loss, and calculations confirm that it does not change the extrapolated end point *τ*_n_.
- Residual gas loss was neglected although, as in [5,6,8–10], the trap cannot be pumped during storage. It will be baked in vacuum and the residual gas composition will be monitored to determine a possible correction to *τ*_n_.

**Fig. 2:**
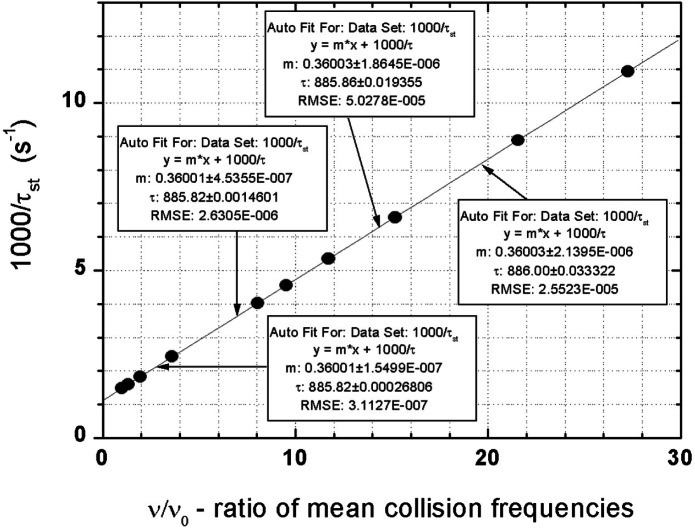
Test of linearity of a plot of inverse storage lifetime *τ*_st_^−1^, versus relative wall collision rate *ν/ν*_0_. For the largest trap volume *V*_max_, *ν*_0_ = 51.9 Hz. The *τ*_st_^−1^ -values are mean values for the storage interval from *t*_1_ = 300 s to *t*_2_ = 2300 s (for the largest volume). The collision rates are mean values for the same time interval. The windows show results of linear fits for subgroups of points (counting from the left): bottom: 1–4; left: 1–5; top: 1–10; right: 7–10. Extrapolated *y*-axis intersections agree within 0.3 s with the value *τ*_n_ = 886 s used in all calculations. The error ranges reflect residuals and do not include counting statistics.

The most important result is: Due to constant trap surface and application of the “scaling technique” for different trap volumes *V*, the dependence of UCN storage loss rate *τ*_st_^−1^ *versus* collision rate *ν* becomes a straight line. The same is true if we plot the mean *τ*_st_^−1^ values from (2) vs <*ν* >, the mean values for *t*_1_ and *t*_2_. Here the mean values are taken for the interval from *t*_1_ = 300 s to *t*_2_ = 2300 s (and scaled down for *V* < *V*_max_). The high degree of linearity is shown in Fig. 2 where linear fits for different subgroups of points for volumes in the range *V*_max_/*V* = 1 to 27 give *τ*_n_ extrapolations within 0.5 s of the initial input value 886 s. Even the four low-volume points farthest from the *y*-intersection (window on right) show no deviation exceeding the anticipated accuracy of the proposed *τ*_n_ experiment. Using a wall loss model with a velocity dependence of losses very different from the step function model, and adding gaps, we obtained the same degree of linearity.

In contrast to a plot vs *γ* (as for method (b)), calculation of collision rates *ν* does not require a specific reflection loss model. However, it does require knowledge of the UCN spectrum and its change during a cycle, which is a difficult experimental task. To approximate *ν* by a quantity based entirely on measured quantities we use the definition of inverse mean free path *λ*^−1^ = *Nν*/Φ = *S*/4*V* where the integral flux is Φ = *N* < *v* > and the velocity is averaged over volume and spectrum. Since the trap surface area *S* is constant, *λ*^−1^ ∼ *V*^−1^ and the collision rate can be expressed as

$$v\sim \Phi /NV\sim \gamma_{e}.$$ (3)

The first proportionality is exact. The second form is a close approximation in terms of loss-corrected efflux rate *γ*_e_ = *γ*_et_ − *τ*_n_^−1^ − *τ*_w_^−1^ = *γ*_et_ − *τ*_st_^−1^, where *γ*_et_ is the initial efflux rate. *γ*_e_ can be represented by directly measurable quantities: total counts *N*; measured storage lifetimes; and count-rates (s^−1^) right after opening the UCN valve at the end of storage times *t*_1_ and *t*_2_. A plot of *τ*_st_^−1^ vs *γ*_e_/*γ*_e0_ shows the same degree of linearity as the plot vs *ν/ν*_0_ in Fig. 2. However, in practice the measurement of time-dependent efflux count-rates may be complicated by the guide section between valve and detector, which gives rise to time delay and losses.

## 5. Conclusions

A new “accordion-type” UCN storage system with low-loss “low temperature Fomblin” coating is proposed. Analysis suggests its suitability for a neutron lifetime experiment with precision < 1 s. This is mainly due to the fact that the trap surface area and its distribution over height remain constant while the volume is changeable in a wide range. Combination with the “scaling technique” of Ref. [5] ensures that the extrapolation from measured storage lifetimes to the lifetime for β-decay is almost exactly linear and therefore reliable. This is true under gravity and for any energy dependence of wall losses and any spectrum of stored UCN.
